# Perception of body size and body dissatisfaction in adults

**DOI:** 10.1038/s41598-021-04706-6

**Published:** 2022-01-27

**Authors:** Wojciech Gruszka, Aleksander J. Owczarek, Mateusz Glinianowicz, Monika Bąk-Sosnowska, Jerzy Chudek, Magdalena Olszanecka-Glinianowicz

**Affiliations:** 1 Health Promotion and Obesity Management Unit, Department of Pathophysiology, Medical Faculty in Katowice, Medical University of Silesia, Katowice, Poland; 2 Pathophysiology Unit, Department of Pathophysiology, Medical University of Silesia, Medyków Street 18 20, 40-752 Katowice, Poland; 3 Department of Psychology, Chair of Social Sciences and Humanities, School of Health Sciences in Katowice, Medical University of Silesia, Katowice, Poland; 4 Department of Internal Medicine and Oncological Chemotherapy, Medical Faculty in Katowice, Medical University of Silesia, Katowice, Poland

**Keywords:** Psychology, Risk factors

## Abstract

Self-perception of body size seems to be not always in line with clinical definitions of normal weight, overweight and obesity according to Word Health Organization classification. The effect of self-perception of body size disturbances and body dissatisfaction may be the development of eating disorders, such as anorexia nervosa or binge eating disorder—a major risk factor of obesity development. Therefore, the study aimed to assess separately the perception of weight status and body size as well as body dissatisfaction in adults with normal weight, overweight and obesity. The study included 744 adults (452 women; 35.9 ± 12.4 years; 21 underweight, 326 normal weight, 221 overweight, 176 obese) referred to Metabolic Management Center and volunteers. Body size perception and body dissatisfaction were assessed based on Stunkards’ Figure Rating Scale (FRS). Additionally, participants’ were asked: ‘Do you think you are: underweight/normal weight/overweight/obese?’ to assess perception of weight status. Participants’ weight and height were measured to calculate body mass index (BMI) after completing the FRS. Individuals within the overweight BMI range have rated themselves as underweight (1.4%), normal weight (30.8%) and obese (2.8%). Also individuals within the obesity BMI range have rated themselves as normal weight (2.6%), and overweight (41.6%). Compatibility of self-assessment of weight status with BMI category according to the measured values was moderate—Kappa coefficient was 0.59 (95% CI: 0.54–0.64). Underestimation of weight status was significantly more common among men than women. There were statistically significant differences in the distribution of body dissatisfaction according to the weight in both women and men. Normal-weight subjects less often than overweight and obese were dissatisfied with their own body size. The degree of body dissatisfaction was greater among women than among men. Adults subjects frequently underestimate their own weight status and body size. Women with overweight and obesity more often than men are dissatisfied with their own body size.

## Introduction

Body image is conceptualized as a multidimensional construct that includes positive and negative self-perceptions and attitudes (i.e., thoughts, feelings, and behaviours) regarding the body^1,2^. This global term includes subjective, affective, cognitive, behavioural and perceptual processes^3^. Incorrect assessment of body size in patients with bulimia nervosa and anorexia nervosa were confirmed previously^4,5^. Recently Ralph-Nearman et al.^6^ shown that patients with anorexia nervosa demonstrate not only greater differences between their own current and ideal body and body dissatisfaction but also regional perceptual inaccuracy for their own current body than healthy controls. In addition, a systematic review performed by Shagar et al.^7^ showed that body dissatisfaction is an important risk factor for the development of eating disturbances in adolescents. It should be noted, that the relationship between body image and body size is not well known among overweight and obese subjects. The growing epidemic of obesity^8^ indicates the need to extend the assessment of body image disturbances to subjects with overweight what may help to prevent the progression to obesity. Analysis of these disturbances and finding tools, allowing to assess them easily, may provide valuable guidance for its use in clinical practice. The screening of subjects for body image disturbances may select the group requiring psychotherapy. Its implementation may in some of them prevent the development of obesity, while in the obese increase the effectiveness of its treatment.

Body dissatisfaction is defined as negative attitude towards one’s own physical appearance and is the effect of perceived discrepancy between actual body image and the desired ideal body image^9^. In turn, body size perception is defined as the accuracy with which one perceives one’s body size and is referred to as the perceptual component^10^.

Factors contributing to the development of body image disturbances include biological factors like gender, age, race, weight changes and socio-cultural factors^11–14^. Misperception of overweight when individuals is in normal weight or underweight BMI range seems to be more common in women than in men and among Whites individuals than in Blacks or Hispanics individuals^15,16^. Dorsey et al.^15^ observed that normal weight non-Hispanic Whites women are more likely to consider themselves as overweight than non-Hispanic Blacks. However, some authors shown that the racial/ethnic influence on body image does not occur if the analysis is adjusted by BMI^17^. Stock et al.^18^ observed that German students were more likely to perceive themselves as being fatter than their BMI compared with their Lithuanian peers, suggesting the former socialism in Lithuania as different cultural background. Moreover, in another study of university students from 21 countries, despite the general low BMI, many perceived themselves as overweight, trying to lose weight (44% of women, 17% of men) and used diets (14% in women and 3% in men) with large differences between studied countries^19^. Contrary, Wardle et al.^20^ assed body image among university students from 22 countries did not show differences in the perceptions of overweight between regions of the world.

Most societies have nearly always created ideals of beauty and attractiveness, often extremely difficult, if not impossible, to achieve. Traditionally, it was thought that a major role in creating a very attractive, slim figure especially in women in western culture is played by mass media^21^. Viewing highly attractive individuals is thought to lead to a social comparison process, and this process, as proved by a meta-analysis by Myers and Crowther^22^, is associated with higher body dissatisfaction in both women and men. However, it has been suggested that the media does not drive the development of body dissatisfaction but reminds individuals of their already-existent body dissatisfaction^23^. It has also been proposed that the harmful effects of idealized images are limited to women with a higher level of neuroticism^24^. In addition, Patrick et al.^25^ shown that women who base their self-worth on contingencies have a higher impact of appearance-related comparisons on self-perceived attractiveness.

Disturbances in the perception of body size can have extreme effects on human behaviour. Perceiving themselves as having excess body weight in people with normal weight may motivate them to adherence to healthy behaviours such as changes in diet or regular physical activity. However, it may also be the cause of the development of bulimia and anorexia nervosa. While, a person with obesity perceiving their own body size as too big can be a motivator to starting obesity treatment and as too small—may be the reason for not taking obesity treatment. It seems that a similar effect may have the level of body dissatisfaction. A significant body dissatisfaction may be a motivator to take obesity treatment.

Most of the studies described above were conducted in highly selected cohorts, e.g. university students^16–18,20^. Less is known about self-perception of weight status among people in middle and older age. In addition, previous studies frequently didn’t refer to the definitions of weight status recommended by WHO, combining overweight and obesity as one group of “overweight”^16–18^. It should be noted that self-diagnosis of obesity can be supplanted because it is very stressful therefore patients with obesity prefer to identify themselves as being overweight^26^.

There is a growing literature describing various factors influencing perception of body size and body dissatisfaction. However, there is a lack of data from large groups of young and middle-age depending on the weight status. Therefore, the study aimed to assess separately the perception of weight status and body size as well as body dissatisfaction in adults with normal weight, overweight and obesity.

## Methods

Eight-hundreds-twenty-four respondents, aged over 16 years, who referred to Metabolic Management Center in Katowice, Poland (NZOZ "Line") and volunteers were enrolled in the study between June 2010 and August 2011. The volunteers were recruited by co-authors which are physicians in their outpatient clinics. The reasons for visits were various, excluding overweight or obesity. From all subjects written consent to carry out all the procedures included in the study protocol were collected. For participants under the age of 18 years, informed consent was obtained from a parent and/or legal guardian. The study was approved by the Bioethics Committee of the Medical University of Silesia KNW-0022/KB1/136/I/08. The exclusion criteria were: secondary obesity (endocrine disorders like Cushing’s syndrome and genetic disorders such as Turner or Prader–Willi syndromes), history of mental disorders (lifetime bipolar disorders, schizophrenia and current substance dependence), eating disturbances recorded in medical history and race other than White. Due to incorrect filling or missing data in the questionnaire 80 (9.7%) subjects were excluded. Finally, a study group consists of 744 subjects, including 452 women (60.7%) and 292 men (39.3%). The basic characteristics of the study group are shown in Table 1.

**Table 1:** Characteristics of the study group according to gender and weight status.

	Normal weight	Overweight	Obese	*p*
	N = 347	N = 221	N = 176	
Women [%]	68	48.9	61.4	
**Age [years]**	31.9 ± 10.5	36.4 ± 12.1	43.3 ± 12.9	<0.001
Women	33.3 ± 10.9	40.1 ± 11.0	45.0 ± 12.0	<0.001
Men	28.8 ± 8.9	32.9 ± 12.1	40.7 ± 13.9	<0.001
**Education [N/%]**				
Primary	19/5.5	12/5.5	6/3.5	< 0.001
Vocational	17/4.9	12/5.5	23/13.3	
Secondary	167/48.4	147/66.8	114/65.9	
Higher	142/41.2	49/22.2	30/17.3	
**Marital status [N/%]**				
Married	134/39.8	122/57.8	110/65.9	< 0.001
Widowhood	176/52.2	79/37.5	32/19.1	
Divorced	27/8.0	10/4.7	25/15.0	
Offspring [N/%]	142/45.6	110/53.9	125/76.8	< 0.001
Smoking [N/%]	109/31.4	74/33.5	50/28.4	0.56
Alcohol [N/%]	248/74.5	160/72.4	113/64.2	0.15
**Body weight [kg]**	62.4 ± 9.1	78.4 ± 9.6	99.6 ± 16.9	<0.001
Women	58.1 ± 6.5	72.2 ± 6.8	94.3 ± 14.8	<0.001
Men	71.6 ± 6.8	84.3 ± 8.0	107.9 ± 16.8	<0.001
**BMI [kg/m**^2^]	22.2 (20.7/23.7)	27.2 (25.9/28.3)	34.3 (31.5/37.5)	<0.001
Women	21.7 (20.3/23.3)	27.3 (26.0/28.3)	35.6 (31.9/37.6)	<0.001
Men	23.0 (21.6/24.1)	27.1 (25.9/28.1)	34.2 (31.2/37.4)	<0.001
**Waist circumference [cm]**	79.5 ± 7.2	92.0 ± 7.7	110.2 ± 12.8	<0.001
Women	77.6 ± 8.9	89.7 ± 7.3	107.9 ± 12.1	<0.001
Men	83.4 ± 6.3	94.2 ± 7.6	113.8 ± 13.0	<0.001

Mean ± standard deviation or median (lower and upper quartile).

Body weight (without shoes, in light clothing, using the certified electronic RADWAG balance, with an accuracy of 0.1 kg) and height (in an upright standing position, without shoes, with an accuracy of 0.5 cm, using an integral part of RADWAG balance) were measured. Body mass index (BMI) was calculated using the standard formula. Assessment of weight status was based on BMI according to WHO criteria^27^.

**Figure 1:**
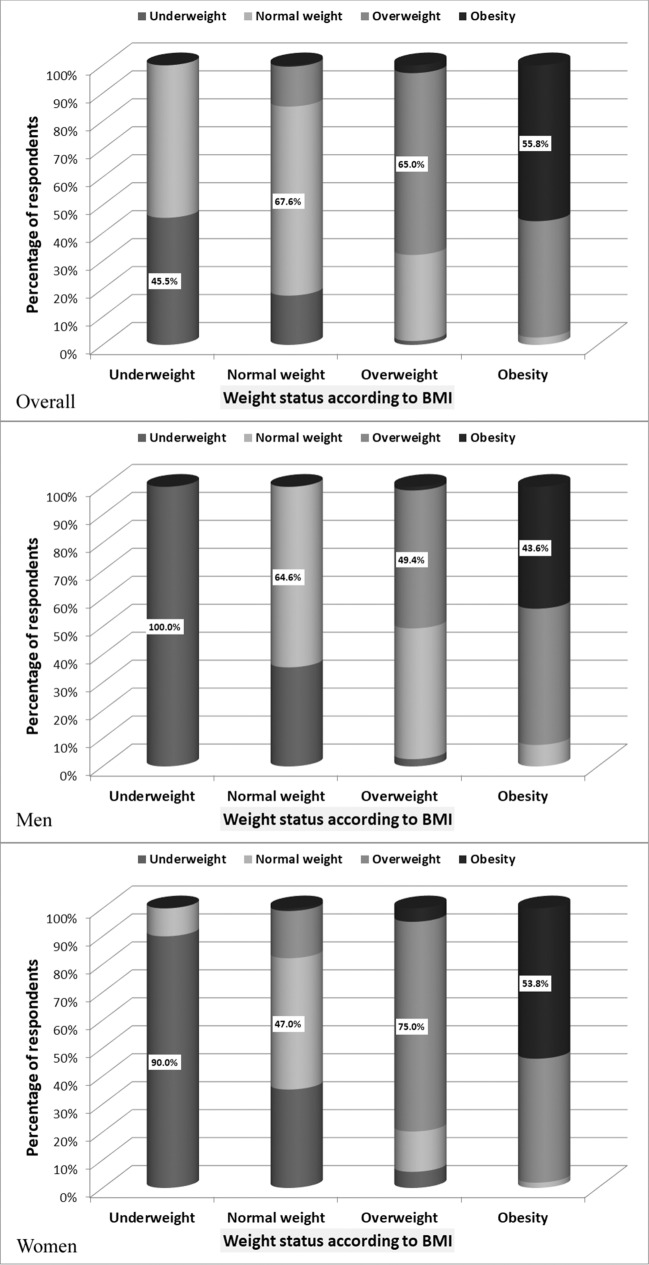
Comparison of compliance between subjective assessment of weight status according to the question (Do you think you are: underweight/normal weight/overweight/obese?) and weight status based on the BMI.

**Figure 2:**
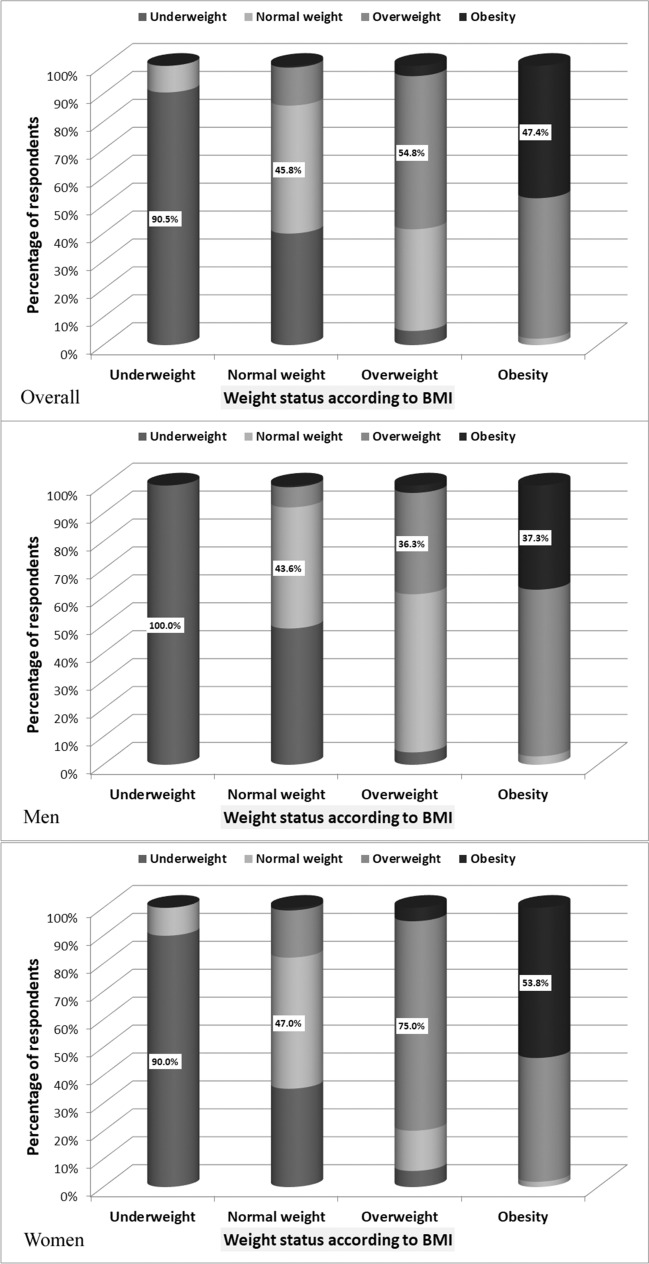
Comparison of compliance between subjective assessment of weight status according to Figure Rating Scale and weight status based on the BMI.

**Figure 3:**
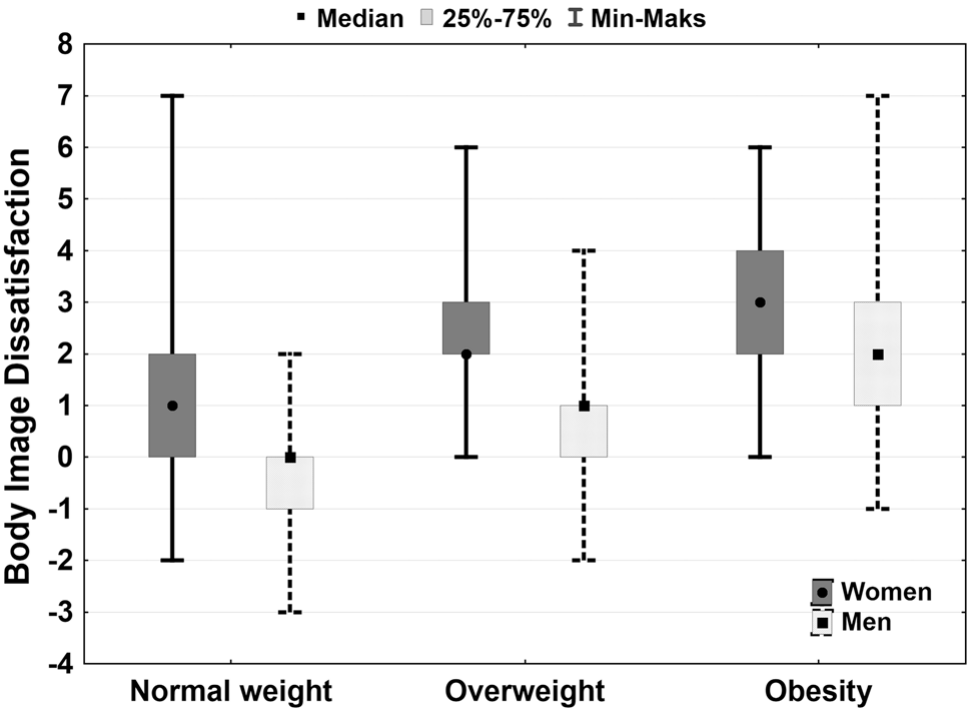
Influence of body weight and gender on body dissatisfaction in groups of men and women.

To assess the perception of weight status, the following question was posed: “Do you think you are: underweight/normal weight/overweight/obese?” This question was added to the study protocol by the authors and it is not a part of Stunkards’ Figure Rating Scale (FRS). As a next step perception of the body size and body dissatisfaction (defined in the study as the discrepancy between figures indicated as currently owned and desirable) were assessed based on FRS. The following instruction was given: “mark the silhouette which is the most similar to yours” and “mark the silhouette which you desire to possess”. This scale has been standardised for use in subjects with obesity and it is recommended by most of the authors. Due to the use of the figures does not need adaptation to the native language of studied subjects^28–30^. FRS is considered a valid and reliable assessment tool in large, diverse populations including women and men^31^. The subjects, according to their gender, were asked to choose male or female figures^29^. The nine figures were divided into four BMI categories, constant with a previous study of similar design (underweight—figures 1 and 2; normal weight—figures 3 and 4; overweight—figures 5–7, obesity—figures 8 and 9)^32^.

### Statistical analysis

Statistical analysis was performed with Statistica 12.0 software (polish version). The results were presented as: mean ± standard deviation for normally distributed data, median and upper and lower quartiles for the data deviate from the normal distribution and the percentages for the data in nominal and ordinal scale. The assessment of distribution was based on the Shapiro–Wilk test.

Comparison of variables in nominal and ordinal scale was carried out using the χ^2^ test (when Cochran conditions are met, regarding the minimum expected value in r × c contingency tables with fixed margins when testing independence with Pearson’s χ^2^ statistic using the χ^2^ distribution) or the Jonckheere-Terpstry test otherwise. Compatibility between BMI classification and subjective assessment of the weight status based on measured values or according to the FRS were evaluated with the Kappa coefficient. Compatibility between figures owned and desirable was performed with the Kołmogorow–Smirnow test, as we compare the distribution of the participant’s given answer and distribution of owned figure. The results were considered as statistically significant with a *p* value of less than 0.05. All tests were two-tailed and no imputation of data was done.

### Ethics approval

All procedures performed in studies involving human participants were in accordance with ethical standards of the institutional research committee and with the 1964 Helsinki declaration and its later amendments or comparable ethical standards.

### Consent to participate

Informed consent was obtained from all individual participants or their legal guardians included in the study.

## Results

### Perception of weight status

Four-hundreds-seventy-seven subjects (64.1%) of all the respondents assessed their weight status, answering the question: ‘Do you think you are: underweight/normal weight/overweight/obese?’. Among them, 9.2% assessed themselves as underweight, 40.9% as normal weight 35.6% as overweight and 14.3% as obese. The remaining respondents did not answer this question. Compatibility between self-assessment of weight and nutritional status assessed according to WHO criteria based on BMI was moderate (Fig. 1). Kappa coefficient was 0.59 (95% CI: 0.54–0.64).

Only 63.5% of respondents correctly assessed their own weight status. Among those with normal weight 17.6% assessed themselves as underweight, 14.3% as overweight, and 0.5% as obese. Among overweight 1.4% have rated themselves as underweight, 30.8% as normal weight, and 2.8% as obese. Among obese 2.6% have rated themselves as normal weight and 41.6% as overweight. In the group of subjects who incorrectly assessed their own weight status men significantly more often than women underestimated weight status (χ^2^ = 52.1, *p* < 0.001).

### Perception of body size

In FRS assessments, 21.7% respondents indicated as currently owned underweight figures (figures 1 or 2), 31.7%—normal weight figures (figures 3 or 4), 34.1%—overweight figures (figures  5 or 6 or 7) and 12.5%—obese figures (figures 8 or 9). Compatibility between self-assessment of body size in FRS and classification of nutritional status assessed according to WHO criteria based on BMI was weak (Fig. 2). The Kappa coefficient was 0.31 (95% CI 0.26–0.36).

Only 49.5% of respondents correctly assessed their body size in FRS. Among those with normal weight 39.8% assessed themselves as underweight, 13.7% as overweight, and 0.7% as obese. Among overweight 4.9% have rated themselves as underweight, 35.7% as normal weight, and 4.6% as obese. 2.7% of subjects with obesity have rated themselves as normal weight and 49.9% as overweight. In the group of subjects assessed in FRS their body size improperly, men significantly more often than women underestimated body size (χ^2^ = 19.9, *p* < 0.001).

### Body dissatisfaction

As the desirable figure, most of the respondents (31.6%) indicated Fig. 3, the least—figure 8 and 1 (respectively 0.4% and 0.3%). 8.9% of respondents wanted to have a figure bigger than they have currently, 65.7%—smaller, 25.4%—the same. The distribution of body dissatisfaction is shown in Fig. 3. Comparisons of distributions by the Kolmogorov–Smirnov test showed statistically significant differences between owned and desirable body size (D = 0.31, *p* < 0.001).

Using the Jonckheere-Terpstry test (due to the unbalanced distribution of data and failure to meet Cochran condition), statistically, significant differences were noted in the distribution of satisfaction with the body size according to the weight status both in women (J = 13.36, *p* < 0.001) and men (J = 12.62, *p* < 0.001). Subjects with normal weight less frequently than with overweight and obesity were dissatisfied with their own body size. The degree of dissatisfaction with body size was greater among women than among men—Fig. 3.

## Discussion

Our study showed, that a large percentage of adults underestimate their own weight status and body size. The perception was assessed separately using two methods, and both confirmed a tendency for underestimation regardless of the BMI category of weight status assessed by the WHO criteria. Of note, the male gender was associated with a greater frequency of underestimation of both weight status and body size. These results are in accordance with the previous study performed in a young Mexican population, assessing weight status^33^. However, our results extend this observation to middle and older age people. It should also be noted that a tendency to underestimate body weight along with overestimation of height were previously described in adults^34^. The high frequency of the underestimation of body size among men was also previously described in a population of the former European Union^35^.

In our study, 41.6% of subjects with obesity underestimated their own weight status. Thus, individuals in this study subgroup more realistically assessed their own weight status than in the cohort enrolled by Howard et al.^36^, where 65% of subjects with obesity underestimated their own weight status to overweight. Moreover, in our study only 49.9% of subjects with obesity assessed their own body size as overweight and 2.7% as normal weight. However, these studies do not explain the reasons for this underestimation. The other published studies suggested that it may be associated with increased incidence of overweight and obesity in the population and comparing yourself to ‘average’ person in their surroundings^8,37^. This phenomenon is also useful to explain by ‘visual normalization theory’—the exposure to obesity results in the normalization of larger body size in terms of self-perceived weight status, children’ weight status perceived by parents and perceiving weight status in others^38^. Moreover, Puhl et al.^39^ have shown that less negative terms such as 'weight problem' and 'unhealthy weight’ motivated more to lose weight than the diagnosis of overweight or obesity. Furthermore, as was mentioned above self-diagnosis of obesity is very stressful therefore patients with obesity prefer to identify themselves as being overweight^26^. Of interest, Robinson et al.^26^ discussed extensively the question ‘it is better to know or not know’ that you are overweight or obese. It should be noted that ‘not to know’ was a barrier to taking treatment of obesity^40^. Notwithstanding, Essayli et al. found that informing people that they are overweight resulted in the increased negative affect and lower self-esteem^41^. Thus, both from psychological and medical points of view, this is a very difficult problem. For a patient, the diagnosis may become a stigma, while failure to diagnose and the lack of treatment may result in the development of obesity complications. Interestingly, Kim et al. observed that a worse profile of cardio-metabolic markers in adults identifying themselves as overweight or obese than in those who did not identify themselves like that^42^. The experimental data of long-term mental and physical consequences of self-identification as overweight and obese are scarce and necessitate further analyses.

In the present study, only 63.5% of subjects that answered the question ‘Do you think you are: underweight/normal weight/overweight/obese?’ correctly assessed their own weight status. It should be noted that as much as 35.9% of study subjects did not answer this question at all. We cannot exclude that it might be caused not only by the disturbances in the assessment of their own weight status and body size but also by different terminology used to name its (for instance: ‘overweight’, ‘about right’, ‘underweight’^16^; ‘much too fat’, ‘too fat’, ‘just right’, ‘too thin’, ‘much too thin’^17^; ‘very overweight’, ‘slightly overweight’, ‘about right’, ‘slightly underweight’, ‘very underweight’^20^). In our study, we used two forms of weight status assessment—verbal and pictorial, and we obtained the selection of body size figures in all subjects included in the analysis. It confirms that the pictorial self-assessment of weight status may avoid more drastic verbal self-classification as being overweight or obese. However, it has also been shown that subjects with obesity more often perceive obesity in others than in themselves^43^. These disturbances in the perception of own body size may be the reason for not treating obesity. This hypothesis was confirmed by Duncan et al. that found that self-perception of body size is more motivating to taking measures to change your body weight than the medical diagnosis^44^. This can also be the cause of drop-out in professional obesity treatment programs^45^. Therefore, to increase the effectiveness of the treatment of obesity, there is a need to analyse the perception of weight status and body size perception in the subjects with overweight and obesity.

It should also be mentioned that in our study 17% of subjects with normal weight underestimated the body size and 14% overestimated it. The percentage of subjects who overestimated the body size was comparable to that observed by Paeratakul et al.^16^. In this study, 18% of normal weight subjects reported that they were overweight. The first case may be associated with the risk of development of overweight and the second with the risk of development of anorexia nervosa or bulimia nervosa. In addition, as has been shown previously, disturbances in the body size perception may be the cause of the development of depressive symptoms^46^.

Only 25% of all respondents were satisfied with their own body size. Of the remaining respondents, 9% wanted to have a higher body weight and 66% lower than currently owned. The percentage of people who desire to have a lower body weight in the present study was similar to that observed in previously published studies^47^. The desire to have a higher body weight may be due to the fact that men want to have a more muscular figure^48^ or to the old belief that the obese can survive in harsh conditions or that obesity is a symbol of wealth and power^49^. It is worth mentioning that recent studies indicate a growing desire for larger, but more muscular body figures also among women^50^. This desire for more muscularity, as recently suggested by Uhlmann et al.^51^, can play a protective role against eating disorder symptomatology. However, these aspects were not assessed in our study.

In line with previously published studies^52,53^ in our study subjects with normal weight, less often than with overweight and obesity, did not accept their own body size and the dissatisfaction with body size was more frequent among women than among men. As has been shown recently, dissatisfaction with body size may be an important risk factor for depressive symptoms development, especially in women^54–56^. It has also been suggested that men have a greater tendency to a more positive perception of their body, which according to the illusion theory, saying that the positive illusion may be the basis of good psychological functioning^57^, protects obese men against the onset of depressive symptoms. Although in our study we confirmed that men are more satisfied with their body size, we cannot confirm the impact of this satisfaction on their well-being.

The strength of the study is the use of two methods of assessment of weight status and body size. In addition, the most of previously published studies^16–18,20^ used self-reported body weight and height to calculate BMI and assessment of weight status while in our study both these parameters were measured by physicians. Other important advantages of our study were the inclusion of middle and older age subjects and a large group of subjects with obesity. While the previous studies assessed mainly young adults with normal weight^16–18,20^. The assessment of subjects with overweight may be useful in determining obesity prevention programs taking into account the elements of psychotherapy aimed at making self-esteem real.

However, the presented study has some limitations such as the small number of underweight respondents. Therefore, we omitted this subgroup in the discussion to avoid misinterpretation of the data. Another limitation is that only 64.1% of the respondents assessed their weight status in the questionnaires, answering the question: ‘Do you think you are: underweight/normal weight/overweight/obese?’. The reasons for that remain unknown. One of the explanations can be a fact that we used medical definitions of weight status, not colloquial language terms, like other authors^16,17,20^. It could make the distinction difficult for some respondents so that they have omitted this question. The main limitation of our study is the solitary use of FRS for the assessment of body size perception because this scale does not allow for the evaluation of individual areas of the body that could be large or small^50,58,59^. However, FRS is one of the validated scales in subjects with obesity. Authors of the scale^29^ did not divide figures into BMI categories and does not include precise, incremental graduations between presented figures, currently perceived as a standard^27^. In our study, four BMI categories of the figures were used in line with previous similar design studies^32,60^. Currently, available figure rating scales such as the Female Body Scale (FBS) or the Male Body Scale (MBS) introduce also an assessment of both adiposity and muscularity body size, dissatisfaction for both women^50^ and men^59^. In addition, the present study does not include the assessment of positive aspects of body image that cannot be viewed as simply the lack of negative features^1,61^. The assessment of positive aspects of body image among subjects in middle and old age will be a continuation of our research.

In conclusions, in our sample adult subjects frequently underestimated their own weight status and body size. This problem is especially frequent among subjects with obesity. These subjects seeing themselves as overweight or normal weight will not undertake obesity treatment. It may also be difficult to accept the diagnosis made by the doctor. It seems that self-assessment of weight status and body size should be incorporated into the daily clinical management of people with obesity. The assessment of these parameters may also be important in people with overweight as part of the prevention of obesity. Further studies research should be carried out on the impact of psychotherapy on changes in perception of weight status and body size and in consequence on the effectiveness of obesity treatment. In addition, our study showed that women with overweight and obesity are more often than men dissatisfied with their own body size. It is worth investigating whether this dissatisfaction motivates or demotivates obesity treatment. Moreover, it would be interesting to investigate whether this dissatisfaction affects the incidence of depressive disturbances among women with overweight and obesity.

## Data Availability

The datasets used and/or analyzed during the current study are available from the corresponding author on reasonable request.
